# Finding the Needle in the Haystack: Can Natural Language Processing of Students’ Evaluations of Teachers Identify Teaching Concerns?

**DOI:** 10.1007/s11606-024-08990-6

**Published:** 2024-08-21

**Authors:** C. Jessica Dine, Judy A. Shea, Caitlin B. Clancy, Janae K. Heath, William Pluta, Jennifer R. Kogan

**Affiliations:** Perelman School of Medicine at the University of Pennsylvania, Philadelphia, PA USA

**Keywords:** Student evaluations of teaching, Natural language processing, Teaching quality

## Abstract

**Background:**

Institutions rely on student evaluations of teaching (SET) to ascertain teaching quality. Manual review of narrative comments can identify faculty with teaching concerns but can be resource and time-intensive.

**Aim:**

To determine if natural language processing (NLP) of SET comments completed by learners on clinical rotations can identify teaching quality concerns.

**Setting and Participants:**

Single institution retrospective cohort analysis of SET (*n* = 11,850) from clinical rotations between July 1, 2017, and June 30, 2018.

**Program Description:**

The performance of three NLP dictionaries created by the research team was compared to an off-the-shelf Sentiment Dictionary.

**Program Evaluation:**

The Expert Dictionary had an accuracy of 0.90, a precision of 0.62, and a recall of 0.50. The Qualifier Dictionary had lower accuracy (0.65) and precision (0.16) but similar recall (0.67). The Text Mining Dictionary had an accuracy of 0.78 and a recall of 0.24. The Sentiment plus Qualifier Dictionary had good accuracy (0.86) and recall (0.77) with a precision of 0.37.

**Discussion:**

NLP methods can identify teaching quality concerns with good accuracy and reasonable recall, but relatively low precision. An existing, free, NLP sentiment analysis dictionary can perform nearly as well as dictionaries requiring expert coding or manual creation.

## INTRODUCTION

Recognition of faculty with teaching quality concerns is important to optimize the educational environment for learners and identify faculty who are struggling with teaching for faculty development and coaching.^1–6^ Analyzing student evaluations of faculty teaching (SET) is one way to pinpoint teaching quality issues.^1,2,7^ SET typically involve rating teaching effectiveness on a numerical scale and providing comments.^2^ While numerical ratings can quickly flag outliers (including through evaluation management software programs), they might not catch subtle concerns, necessitating a manual review of comments. However, evaluating teaching concerns in narrative comments poses challenges. First, manual review is time-intensive. Second, readers need to have a shared mental model to define which words and phrases suggest quality concerns. Third, there is necessary “pre-review” work, such as blinding of learner, faculty, specialty, and rotation.

As a possible solution, natural language processing (NLP) is increasingly being used in medical education to identify linguistic patterns in narrative evaluations.^8–12^ Compared to human raters, NLP could improve consistency, reduce time, identify potentially biased language, and improve ability to provide feedback.^9–17^ We are unaware of research exploring the feasibility of using NLP methods to identify teaching quality concerns in SET. The overarching goal of this study was to determine if NLP techniques could be used to screen SET comments to accurately identify evaluations with teaching quality concerns. A second goal was to compare the accuracy between an existing [free] NLP dictionary to de novo dictionaries manually created by medical education experts for the identification of teaching quality concerns in SET.

## SETTING AND PARTICIPANTS

The Perelman School of Medicine at the University of Pennsylvania has an integrated Teaching Evaluation Database that collects SET across undergraduate medical education, graduate medical education, and biomedical graduate studies.^18^ Since 2017, SET include a question asking learners to rate the overall quality of teaching on a 5-point scale (0 = falls below expectations; 4 = far exceeds expectations). There is a space for optional comments.

From our evaluation database, we extracted all SET of faculty supervisors completed by medical students, residents, and fellows on clinical rotations between July 1, 2017, and June 30, 2018. The dataset included 11,850 evaluations with comments from over 2000 faculty. The analytic dataset included learner level, faculty department, and rank. All SET were de-identified prior to analysis by the Office of Evaluation and Assessment using an initial computerized de-identification program, followed by manual review. We excluded incomplete evaluations (*n* = 26; 0.23%) from the analysis.

## PROGRAM DESCRIPTION

### Identifying Teaching Quality Concern

Three expert raters (CC, JH, JK) independently coded 100 SET for the presence or absence of a teaching quality concern and then reviewed and discussed discrepancies. For the dataset, the expert raters coded each comment as either “1” or “0,” signifying that there was a teaching quality concern present or not, respectively, which served as the outcome for subsequent analysis. These authors then independently coded 300 additional comments to determine the interrater reliability prior to additional coding. Given a kappa of 0.90 from this initial review, authors each coded 33% of the remaining comments from the entire data set; 500 randomly selected comments in the entire data set were triple-coded to ensure ongoing agreement. The final kappa was 0.87.

### Creating NLP Dictionaries

Three NLP dictionaries were created for the analysis, the *Expert Dictionary*, the *Qualifier Dictionary*, and the *Text Mining Dictionary*.

For the creation of the initial dictionary, two authors (JK, JS) independently reviewed 200 randomly selected SET comments with low teaching quality to identify words or phrases commonly used to describe poor teaching quality (kappa was 1.00). To identify additional words and phrases associated with poor teaching quality, these same authors reviewed 231 comments from SET with numerical ratings of 0 or 1, in addition to all comments for the 10 faculty with the lowest average numerical ratings (total 83 comments). They iteratively discussed their word lists to reach consensus. This word list became the Expert Dictionary.

While creating the Expert Dictionary, we appreciated that comments with teaching quality concerns often included qualifying or hedging words (e.g., “possibly,” “perhaps could,” “should,” “might,” “but,” “however”) or conjunctive adverbs (e.g., therefore).^19^ We created a Qualifier Dictionary based on these words alone.

We then used a specialized NLP software program (Wordstat 8.0 Software) to create an automated Text Mining Dictionary. Text mining is an NLP technique that identifies and quantifies words and phrases associated with a concept or topic.^20^ In this case, the program was used to generate a dictionary of words and phrases frequently identified in comments flagged with teaching quality concerns, based on an initial set of 200 randomly selected SET comments with low teaching quality ratings. The text mining process is similar to the creation of the Expert Dictionary; however, it automates the effort of the faculty experts.^20^

Finally, we employed a pre-existing, freely available WordStat Sentiment Analysis Dictionary.^21,22^ Sentiment dictionaries contain words and phrases associated with different emotions or sentiments (e.g., happy, sad, angry, or excited). The dictionary was designed by combining negative and positive words from the Harvard IV dictionary, the Regressive Imagery Dictionary,^23^ and the Linguistic and Word Count dictionary.^24^ If the Sentiment Analysis Dictionary alone can identify comments containing concerns about teaching quality, this would significantly reduce up-front administrative and faculty resources compared to the other models.

### Data Analysis

The dataset was divided into a training dataset (75% or 8868 comments) and a validation dataset (25% or 2956 comments). Each dataset had an equal distribution of flagged comments indicating a teaching quality concern (9%) and unflagged comments (91%), indicating no teaching quality concern.

Using WordStat, we created multiple predictive models using individual and combined dictionaries to identify comments with teaching quality concerns. WordStat implements the naïve Bayes and K-nearest neighbors algorithms to create initial models from a subset of labeled training data. We then used split sample validation methods to test the predictive models in our validation dataset.

To assess the accuracy of each dictionary on the prediction of poor teaching quality, we compared the model performance for each individual dictionary (Expert, Qualifier, Text Mining, and Sentiment Analysis) as well as the combination of the Qualifier (minimal pre-work required) and the Sentiment Analysis Dictionaries (no pre-work required).^21,22^ For each model, we calculated accuracy, precision, and recall. Accuracy measures how often a model correctly predicts the outcome and is calculated as the number of correct predictions divided by the total number of predictions. Precision measures how many of the predicted positive cases actually represent teaching quality concerns. It is the equivalent of the positive predictive value, calculated as the number of true positive (TP) cases (comment is flagged by NLP and contains a teaching quality concern) divided by the total number of predicted positive cases. Recall measures how well the model identifies comments containing teaching concerns, calculated as the number of comments flagged by the NLP model as containing a teaching concern divided by all comments containing a teaching concern (the sum of TP and false negatives (FN)). We also calculated the percent of comments requiring manual review (Percent Flagged = (TP + FP)/all comments) and missed incidences of poor teaching quality (Percent Missed = FN/all comments) to help differentiate between the practicality of using one or more of the dictionaries.

The University of Pennsylvania Institutional Review Board deemed the study exempt.

## PROGRAM EVALUATION

Table 1 summarizes the dataset. Through manual coding by the expert coders, 1065 (9.01%) of the sample had comments reflecting poor teaching quality. Table 2 summarizes the performance of models using individual and combined dictionaries.

**Table 1:** Characterization of Dataset

	*n* (%)
Total evaluations (*n*, %)	11824
Evaluations with teaching quality concern (*n*, %)	1065 (9.0%)
Evaluations by level of trainee	
Residents/fellows	8856 (74.9%)
Medical students	2968 (25.1%)
Rating on student evaluations of teaching (SET) (mean, std dev)	3.68 ± 0.62
Total faculty (*n* )	2199
Faculty gender (*n*, %)	
Female	943 (42.8%)
Male	1197 (54.3%)
Undisclosed/missing	59 (2.7%)
Faculty race (*n*, %)	
Asian	399 (18.1%)
Black	80 (3.6%)
Hispanic	56 (2.6%)
White	1542 (70.1%)
Multiple	37 (1.7%)
Undisclosed/missing	85 (3.9%)
Faculty department (*n*, %)	
Anesthesiology and Critical Care	189 (8.6%)
Cell and Developmental Biology	1 (0.1%)
Dermatology	49 (2.2%)
Emergency Medicine	75 (3.4%)
Family Medicine and Community Health	35 (1.6%)
Medical Ethics and Health Policy	3 (0.1%)
Medicine	573 (26.1%)
Neurology	113 (5.1%)
Neurosurgery	19 (0.9%)
Obstetrics and Gynecology	73 (3.3%)
Ophthalmology	31 (1.4%)
Orthopedic Surgery	45 (2.1%)
Otorhinolaryngology: Head and Neck Surgery	34 (1.6%)
Pathology and Laboratory Medicine	77 (3.5%)
Pediatrics	437 (19.9%)
Pharmacology	1 (0.1%)
Physical Medicine and Rehabilitation	22 (1.0%)
Psychiatry	102 (4.6%)
Radiation Oncology	34 (1.6%)
Radiology	141 (6.4%)
Surgery	145 (6.6%)

**Table 2:** Performance of NLP Dictionaries

	True positives (TP)	True negatives (TN)	False positives (FP)	False negatives (FN)	Accuracy (TP + TN)/all	Precision (TP)/(TP + FP)	Recall (TP)/(TP + FN)	Percent flagged (TP + FP)/total	Percent missed (FN)/total
Training dataset (75%)									
Expert	522	6791	1275	280	0.82 ± 0.01	0.29 ± 0.01	0.65 ± 0.01	20%	3%
Qualifier	554	5076	2990	248	0.63 ± 0.01	0.16 ± 0.01	0.69 ± 0.01	40%	3%
Text mining	704	7068	998	98	0.88 ± 0.01	0.41 ± 0.01	0.88 ± 0.01	19%	1%
Sentiment	546	6273	1793	256	0.77 ± 0.01	0.23 ± 0.01	0.68 ± 0.01	26%	3%
Sentiment and qualifiers	685	7039	1027	117	0.87 ± 0.01	0.40 ± 0.01	0.85 ± 0.01	19%	1%
Validation dataset (25%)									
Expert	179	2489	108	179	0.89 ± 0.01	0.62 ± 0.02	0.50 ± 0.02	10%	6%
Qualifier	176	1738	955	87	0.65 ± 0.02	0.16 ± 0.01	0.67 ± 0.02	38%	3%
Text mining	175	2127	566	88	0.78 ± 0.01	0.24 ± 0.02	0.67 ± 0.02	25%	3%
Sentiment	149	2103	590	114	0.76 ± 0.02	0.20 ± 0.01	0.57 ± 0.02	25%	4%
Sentiment and qualifiers	202	2342	351	61	0.86 ± 0.01	0.37 ± 0.02	0.77 ± 0.02	19%	2%

The Expert Dictionary model had the highest accuracy (0.89) and precision (0.62), but the lowest recall (0.50) among all models. This corresponds to the Expert Dictionary model correctly predicting 89% of comments (accuracy), with 62% of positive flags reflecting true teaching concerns (precision), and 50% of true teaching concerns flagged by the model (recall). Overall, the Expert dictionary flagged 10% of all comments for further review (TP + FP/all comments), with a 6% miss rate (FN/all comments).

The Qualifier Dictionary had the lowest accuracy (0.65) and precision (0.16), and intermediate recall (0.67), flagging 38% of comments for further review, with a 3% miss rate.

The Text Mining model had intermediate accuracy (0.78), lower precision (0.24), and intermediate recall (0.67), flagging 25% of comments for further review and missing 3% of comments containing teaching quality concerns.

The Sentiment analysis model had intermediate accuracy (0.76), but low precision (0.20) and recall (0.57), flagging 25% of comments for further review with a 4% miss rate.

The combination model (Sentiment + Qualifiers) had similar accuracy (0.86) to the Expert Dictionary, intermediate relative precision (0.37), and the highest recall (0.77) among all models, flagging 19% of comments for further review with a 2% miss rate.

## DISCUSSION

In this study, we explored whether NLP methods could be employed to identify concerns about faculty teaching quality in SET comments and assess the relative performance of models with varying degrees of up-front effort requirements. We found that all the NLP models could be used to identify teaching quality concerns with reasonable accuracy, correctly categorizing comments 65 to 90% of the time. While prior research has shown that NLP can be used to identify biases in SET^25^ and examine vocabulary used in trainee performance evaluations,^10,26^ we believe this is the first study to explore using NLP to screen for poor teaching quality.

In our study, the most resource-intensive model (Expert Dictionary) would lead to the most significant reduction in comments requiring manual review, but missed 6% of comments with concerns. Of note, we found that a combination of generic dictionaries (Sentiment Analysis plus Qualifier Dictionary), requiring less effort to generate and maintain, achieved similar accuracy to the resource-intensive Expert Dictionary Model, correctly categorizing 86% of comments. Employing this generic combination model would reduce the number of comments requiring manual review to 19%, and only miss 2% of comments with concerns. The Text Mining Model, which requires manual creation of an initial training set by flagging comments that suggest poor teaching quality but then automates the dictionary creation, performed intermediately, with 78% accuracy, flagging 25% of comments for review, and missing only 3% of comments with concerns.

Through this work, we offer alternatives for educators who are responsible for program evaluation and/or identifying teachers who might benefit from faculty development to improve their teaching quality.^6,6^ In our study, all NLP approaches except the Expert Dictionary, which required significant time investment from three faculty reviewers, greatly reduced the scope of the review task. As an example, at our institution, the current practice is for all comments to be manually reviewed by program evaluation staff and faculty, accounting for approximately 60 h of work for review of the approximately 12,000 comments in this database. The combination of generic models (Sentiment Analysis plus Qualifier Dictionary), which requires minimal up-front effort, could potentially reduce the number of comments requiring manual review from 12,000 to 2280, decreasing manual review time to approximately 11 h, representing significant time savings. We estimate the time to create the Expert Dictionary by reviewing a smaller subset of comments is 15 h. The Text Mining Model requires up-front labeling of comments with teaching concerns to train the model but does not require the time associated with building the dictionary. While faculty time is required up-front to create models and longitudinally for iterative review, this approach provides marked time savings for educators involved in program evaluation. Time and effort saved in streamlining and semi-automating the identification phase could be devoted towards faculty development efforts to improve faculty performance, which often requires extensive resources.^27,28^

Importantly, while this process incurred substantial time savings in the review of teaching quality by our program evaluators, this process may have a less substantial impact on time savings for SET reviews in other settings, such as promotions committee, teaching awards, and curricular design. This process could offer important opportunities for the identification of faculty eligible for teaching awards, bonuses, and aid in faculty development. Additional evaluation of the role of this technology in these domains is warranted.

There are several limitations. First, while our study used SET to identify teaching quality concerns, there is important literature identifying myriad biases with SET, based on teacher demographics such as gender, age, and level of attractiveness.^29–34^ It is critical that an NLP-guided approach serve within a larger iterative review and not in isolation, for any higher-stakes decisions in faculty development. Generalizability may be limited given this study took place at a single large academic medical center. Institutions may have unique norms related to evaluation.^35–38^ While the Expert Dictionary was created by two faculty members with extensive experience in assessment, other individuals might be able to create a more comprehensive dictionary. Given that evaluations and language patterns change over time,^39,40^ any NLP dictionary needs to be re-validated over time (which is particularly relevant with this study, noting the dataset was from 2017 to 2018). We anonymized the data set so it is unclear how many unique faculty were identified that would not have been found using just ratings. We focused on SET from clinical rotations; it is not certain if the findings would apply to other learning venues.

In conclusion, NLP techniques can be used to identify teaching quality concerns in clinical rotations from SET. Existing dictionaries such as sentiment dictionaries may be as effective as manually created dictionaries.
